# Varaps: a python package for estimating SARS-CoV-2 lineages proportions from pooled sequencing data (ANRS0160)

**DOI:** 10.1186/s12859-025-06299-7

**Published:** 2025-11-23

**Authors:** El Hacene Djaout, Nicolas Cluzel, Vincent Marechal, Gregory Nuel, Marie Courbariaux

**Affiliations:** 1 LPSM, Sorbonne university, Paris, France; 2 SUMMIT, Sorbonne university, Paris, France; 3 Sorbonne Université, Inserm, Centre de Recherche Saint-Antoine UMRS 938, 75012 Paris, France; 4 OBEPINE GIS, Paris, France; 5 Université Paris-Saclay, INRAE, AgroParisTech, 78350 Gabi, Jouy-en-Josas France

**Keywords:** SARS-CoV-2, Variant proportions, Pooled sequencing, Python package

## Abstract

****Background**:**

Wastewater-based epidemiology has been investigated as a very effective way of monitoring SARS-CoV-2 variants. This can be achieved through accurate lineage deconvolution of wastewater sequencing data. Variants Ratios from Pooled Sequencing (VaRaPS) is a Python package designed for this purpose, utilizing pooled sequencing data and lineage mutation profiles to estimate their proportions.

****Results**:**

VaRaPS re-implements core algorithms from the literature, achieving significant improvements in computational speed and efficiency. Comparative analyzes with simulated and synthetic data sets demonstrate its superior performance in lineage prevalence estimation, underscored by its user-oriented design for broader accessibility.

****Conclusions**:**

By improving speed and accuracy in SARS-CoV-2 variant analysis, VaRaPS offers valuable insights into viral evolution, supporting ongoing surveillance efforts in the post-pandemic landscape.

## Background

Estimating the dynamics of variants of concern is, to date, a key issue for monitoring the SARS-CoV-2 epidemic [1, 2]. Wastewater-based epidemiology allows macro-epidemiological monitoring of these dynamics [3, 4]. A direct approach consists of quantifying the presence of lineages one by one, for instance by digital RT-PCR (Reverse-Transcriptase Polymerase Chain Reaction) targeting a signature mutation of each monitored lineage [4].

Whole Genome Sequencing (WGS) of SARS-CoV-2 offers new perspectives albeit at a higher cost (still much lower than sequencing of individual clinical samples). A first approach to estimate lineages prevalence from this data consists in focusing on signature mutations frequencies [5], or even co-occurrences of those mutations [6], supposing that the frequency of the corresponding lineages should be close to the relative frequency of its signature mutations. However, other information is available in WGS results, such as the prevalence of passenger mutations and mutations common to several lineages [7].

Since a wastewater sample is effectively a pool of viral genomes originating from many individuals, our task resembles other mixture-deconvolution problems that have already been tackled in genomics.

A closely related example is the quantification of transcript isoforms in RNA-seq, where one observes short reads that can align to several splice variants of the same gene. Tools such as Cufflinks, RSEM, kallisto and Salmon model the read counts as originating from a mixture of known isoforms and estimate their relative abundances with expectation–maximisation (EM) or variational inference [8–11].

Additionally, deconvolution tools originally developed for estimating haplotype frequencies in pooled samples can be adapted to estimate the prevalence of known lineages of interest in wastewater treatment plant samples (WWTPs) [12, 13].

Our lineage-prevalence estimation problem has a similar statistical structure: sequencing reads (or mutation counts) are generated by a mixture of known lineage haplotypes, and we seek the mixing proportions.

Let *M* be a lineage mutation profile built from Global Initiative on Sharing Avian Influenza Data (GISAID) data [14] with mutations in rows, lineages in columns, and each cell $M_{ij}$ corresponding to the observed frequency of mutation *i* in lineage *j*. In other words, $M_{ij}$ indicates how often mutation *i* appears in the genetic sequences of samples belonging to lineage *j*. The matrix *M* is in $\mathbb {R}^{n \times m}$, where *n* is the number of mutations and *m* is the number of lineages. Let $\varvec{\pi } \in {\mathbb {R}^{m}}$ be the vector of lineages prevalence in the sample, to be estimated. A direct approach to estimate $\varvec{\pi }$, chosen by [12], consists in minimizing the $L_1$ norm between the expected mutation profile in the sequenced data, $M \varvec{\pi }$, and the observed mutation profile, *f*:

1 $$\begin{aligned} \hat{\varvec{\pi }} = \underset{\varvec{\pi }}{\textrm{argmin}} \Vert M \varvec{\pi }-f\Vert _1 \end{aligned}$$

where $f \in \mathbb {R}^{n}$ is a vector that contains the frequency of each mutation in the sample (from WGS). This minimization is generally done numerically and a transform of $\varvec{\pi }$ is considered to ensure it is positive and sums up to 1.

In the method Freyja [15], weights corresponding to the logarithm of the sequencing depth at mutation positions are added:

2 $$\begin{aligned} \hat{\varvec{\pi }} = \underset{\varvec{\pi }}{\textrm{argmin}} \sum _i \log ( d_i+1) \Vert M \varvec{\pi }-f\Vert _1, \end{aligned}$$

where *i* is the mutation index and $d \in \mathbb {R}^{n}$ is the sequencing depth, with $d_i$ the sequencing depth at the position of mutation *i*. The logarithm transform here aims at tackling very local and large sequencing depths that often occur in sequencing data and result in a disproportional impact of mutations located there.

The Lineage deComposition for SARS-CoV-2 (LCS) method [16] relies on a probabilistic mixture model where the sequencing depth corresponds to a number of random observations. In this model, each observation is considered independently and can be assigned to a different lineage. The associated log-likelihood is computed and numerically maximized with respect to the $\varvec{\pi }$ parameters:

3 $$\begin{aligned} \hat{\varvec{\pi }} = \underset{\varvec{\pi }}{\textrm{argmax}} \prod _{i} \left( {\begin{array}{c}d_i\\ y_i\end{array}}\right) (M_i \varvec{\pi })^{y_i} (1 - M_i \varvec{\pi })^{d_i - y_i} \end{aligned}$$

where $d_i$ is the total number of observations for mutation position *i* in the sample (sequencing depth at the position of mutation *i*), $y_i$ is the number of observations of mutation *i*, and $M_i \varvec{\pi }$ represents the expected frequency of mutation *i* given the lineage proportions $\varvec{\pi }$.

VirPool and other equivalent methods [13, 17, 18] extend this model by considering that all the observations located on the same read come from the same lineage, thus fully using the available information through a mixture model at the read-level. There algorithms find $\varvec{\pi }$ maximizing a Likelihood of the form:

4 $$\begin{aligned} \hat{\varvec{\pi }} = \underset{\varvec{\pi }}{\textrm{argmax}} \prod _{k} \sum _{j} \pi _j \prod _{i} M_{ij}^{x_{ik}} (1 - M_{ij})^{1-x_{ik}} \end{aligned}$$

where $\pi _j$ denotes the proportion of lineage *j*, and $x_{ik}$ is an indicator that is 1 if read *k* carries mutation *i* and 0 otherwise.

Among these advanced methods, VirPool’s data processing pipeline does not permit properly taking into account information coming from indel-type mutations (insertions are simply ignored, whereas sequencing depth is used as a substitute to consider deletions) [17]. Furthermore, VirPool estimates variant proportions by optimizing its probabilistic model using the L-BFGS-B algorithm which is not the most suitable likelihood maximization algorithm for this type of model. In contrast, the alternative method developed for SARS-CoV-2 strains [18] requires an elaborate pipeline to select candidate strains and employs an avanced Expectation-Maximization (EM) algorithm to handle the computational cost resulting from working at the strain scale.

Although these methods have shown promise in estimating lineage proportions, they have limitations in terms of computational efficiency and data processing capabilities. Freyja and VirPool’s pipelines do not fully account for indel-type mutations, and LCS’s original implementation lacks a sum-to-one constraint. Moreover, the computational cost of LCS and Virpool methods can be prohibitive for large-scale surveillance efforts, particularly when dealing with the massive amounts of sequencing data generated by WGS.

To address these limitations, we have developed VaRaPS (Variant Ratios from Pooled Sequencing), a Python package that streamlines the estimation of SARS-CoV-2 lineage proportions from pooled sequencing data. VaRaPS reimplements the Freyja, LCS and VirPool methods with improved accuracy and computational efficiency, while also providing a unified framework for data processing and variant calling. Using the full information content of the sequencing reads and optimizing key computational steps, VaRaPS enables the rapid and accurate monitoring of SARS-CoV-2 lineages in wastewater samples, facilitating timely epidemiological insights and public health responses.

## Implementation

VaRaPS is a Python package designed to streamline the estimation of SARS-CoV-2 lineages proportions from pooled sequencing data. The package architecture is modular, allowing flexibility in data input, processing, and output. It supports SAM (Sequence Alignment Map), BAM (Binary Alignment Map) and CRAM (Compressed Reference-oriented Alignment Map) file formats, which are commonly used in sequencing data storage and processing [19]. VaRaPS has been successfully tested on data from both nanopore and Illumina sequencing technologies.

### A tailor-made and efficient variant caller

A key feature of VaRaPS is its custom variant calling algorithm, which is specifically tailored to the needs of pooled sequencing data analysis. Unlike other variant calling tools such as GATK [20] and FreeBayes [21], which are designed primarily for individual samples, VaRaPS performs variant calling for each read separately, which is necessary for the estimation of lineages proportions in pooled samples from the most advanced methods.

VaRaPS leverages the information provided by the individual read alignments. To optimize computational efficiency, VaRaPS uses sparse matrices to store and process the mutation data. Sparse matrices allow for memory-efficient storage and rapid manipulation of large, sparsely populated datasets, which is advantageous when dealing with the substantial volumes of sequencing data generated in wastewater-based epidemiology.

The variant calling results are stored in three key output files: mutations_index, Xsparse, and Wsparse. The mutations_index file serves as a legend, mapping each unique mutation to an index. The Xsparse file contains the actual mutation data for each read, represented in a sparse matrix format, where each row corresponds to a read, and the columns indicate the presence or absence of mutations. The Wsparse file stores the frequency of each unique read, allowing efficient data storage and processing.

### An optimized implementation of deconvolution algorithms

In addition to its custom variant calling approach, a core component of VaRaPS is the re-implementation and optimization of the Freyja [15], LCS [16], and VirPool [17] methods for estimating lineages proportions. These re-implementations aim to improve both the accuracy and computational efficiency of these methods.

#### Taking into account sequencing error rate

The re-implemented methods in VaRaPS incorporate an error rate parameter ($\alpha \in [0,1]$) to account for sequencing errors. Let $X_{ik} \in \{0,1\}$ be the indicator variable for the event "Read *k* has Mutation *i*", and let $X'_{ik} \in \{0,1\}$ be the "erroneous" version of $X_{ik}$ with error rate $\alpha$, representing what we actually observe. The error rate $\alpha$ is defined as:

$$\begin{aligned} \alpha = \mathbb {P}\left( X'_{i k}=1 | X_{i k}=0\right) = \mathbb {P}\left( X'_{i k}=0 | X_{i k}=1\right) \end{aligned}$$

In other words, $\alpha$ represents the probability of observing a mutation where there is none, or conversely, not observing a mutation where one exists. This error can arise from environmental factors or sequencing errors.

Let *M* be the original lineage mutation profile, where $M_{ij}$ is the probability of observing mutation *i* in a read originating from lineage *j* in the absence of sequencing errors. The error rate is incorporated into a modified lineage mutation profile $M'$, calculated as follows:

$$\begin{aligned} M'_{ij}&=\mathbb {P}(X'_{ik}=1|Z_{jk}=1)\\&=\mathbb {P}(X'_{ik}=1,X_{ik}=1|Z_{jk}=1)+ \mathbb {P}(X'_{ik}=1,X_{ik}=0|Z_{jk}=1)\\&=\mathbb {P}(X'_{ik}=1|X_{ik}=1,Z_{jk}=1) \mathbb {P}(X_{ik}=1|Z_{jk}=1)\\&\quad+ \mathbb {P}(X'_{ik}=1|X_{ik}=0,Z_{jk}=1) \mathbb {P}(X_{ik}=0|Z_{jk}=1)\\&=(1-\alpha ) M_{ij} + \alpha (1-M_{ij})\\&=M_{ij} - 2 \alpha M_{ij} + \alpha , \end{aligned}$$

Where $Z_{jk}$ $\in \{0,1\}$ is, for any read *k*, the indicator latent variable of the event "Read *k* is from Variant *j*".

The error rate $\alpha$ is typically assumed to be around 0.04 for nanopore sequencing and 0.005 for Illumina sequencing [22]. VaRaPS can also calculate and optimize the error rate parameter $\alpha$ to minimize the discrepancy between the observed and expected mutation frequencies, thereby improving the accuracy of lineages proportions estimates.

#### Deconvolution algorithms optimizations

Throughout the implementation, care has been taken to optimize computations, minimizing unnecessary calculations and storing intermediate results when needed to avoid redundant operations. The judicious use of numpy [23] for efficient array operations further enhances the computational performance of VaRaPS.

In our reimplementation of the Freyja method within VaRaPS, we explored various numerical optimization strategies provided by the minimize function from the scipy library in Python. The tested algorithms include the following: Nelder-Mead, Powell, CG, BFGS, Newton-CG, L-BFGS-B, TNC, COBYLA, COBYQA, SLSQP, trust-constr, dogleg, trust-ncg, trust-exact, and trust-krylov.

Additionally, we evaluated different mutation weightings in Freyja’s objective function: by raw sequencing depth, by the logarithm of depth, and without weighting. Extensive empirical analysis across multiple datasets revealed that the combination of the ’TNC’ optimization algorithm and weighting by raw sequencing depth consistently produced the most accurate estimates of lineage proportions. This optimal configuration was therefore retained in the final implementation of Freyja in VaRaPS. On the other hand, to ensure that the estimated lineage proportions sum to 1, we introduce a Lagrangian term in the optimization process in VirPool re-implementation. For Freyja and LCS, we introduced a well-chosen change of variable before passing the input to the optimization function. No such constraint was implemented in the original version of LCS, which could result in proportions summing either lower or higher than one.

In our reimplementation of VirPool, we made significant modifications to enhance its capability in handling indel mutations. Unlike the original VirPool, which ignored insertions and deletions, our version fully incorporates information from both types of mutation. Specifically, we modified the read processing algorithm to explicitly account for indels identified in the CIGAR strings. Inference is then performed using the EM algorithm given as Algorithm 1 in the Appendix.

By combining a custom variant calling algorithm, optimized reimplementations of existing methods, and the incorporation of an error rate parameter, VaRaPS provides a comprehensive and efficient framework for estimating SARS-CoV-2 lineage proportions from pooled sequencing data.

#### Bootstraping and downsampling

Furthermore, we have incorporated a bootstrap feature in VaRaPS to assess the variability of the lineage proportion estimates. The bootstrap is performed on the reads. Specifically, we employ a weighted bootstrap, constructing a sample with the same total number of reads as the original data. The reads are randomly drawn with replacement, according to their relative weights. The bootstrap weights are established by calculating the frequency of occurrence of each read in the resampled data. This bootstrap approach allows for a more robust assessment of the uncertainty associated with the lineage proportion estimates.

VaRaPS provides two distinct forms of “downsampling”, which serve different purposes. First, subsampling to save time in the variant proportion computation step (called "Mode 2" in VaRaPS package): reads are independently and uniformly resampled with replacement from the empirical read distribution to a user-specified target size $n$ (by default the full dataset). This procedure is statistically equivalent to bootstrap resampling, but with $n$ possibly smaller than the original sample size; it is performed entirely in memory without altering the sequence files. Second, file-level downsampling (called "Mode 5" in VaRaPS package), in which new BAM/CRAM files are produced by uniformly sampling reads,without replacement, to a target size, thereby reducing on-disk footprint and I/O; this operation is independent of the bootstrap/subsampling used during deconvolution.

#### Generating custom lineage mutation profiles

VaRaPS further enhances flexibility through an additional functionality (called "Mode 4") which allows users to generate custom lineage mutation profile matrices by specifying desired SARS-CoV-2 lineages, applying filters for mutation frequency and sequence count, and integrating GISAID data with phylogenetic tree information. This feature facilitates customized analyses for emerging variants or alternative viral pathogens, simplifying the adaptation of the package to diverse epidemiological contexts.

## Results

### Validation data

#### Spike-in mixtures

VaRaPS was first validated on a synthetic dataset provided by Karthikeyan et al. [15], which included spike-in mixtures for five prominent SARS-CoV-2 lineages: Beta, Delta, Gamma, A, and Alpha. These mixtures reproduce viral RNA populations found in clinical or environmental samples. This dataset contains 383 BAM files, each with specified proportions of viral lineages, with a read length of 151 bp generated using Illumina sequencing technology. With the actual mixtures used for sequencing being disclosed, this allows for a transparent and precise assessment of the lineage proportion estimation tools integrated within VaRaPS.

#### Simulated data

To complement the wet-lab spike-in mixture, we generated a fully synthetic dataset with known ground-truth abundances. Reads were simulated with SWAMPy [24], which wraps ART Illumina [25].

- **Lineages.** Six representative SARS-CoV-2 lineages were selected: *BA.1*, *BA.2*, *B.1.1.7*, *B.1.351*, *CH.1.1*, and *P.1*. The inclusion of the closely related *BA.1*/*BA.2* pair enables assessment of deconvolution accuracy under minimal inter-lineage divergence.
- **Read simulation parameters.** Default SWAMPy settings were retained: 2 $\times$ 250 bp paired-end reads, empirical HiSeq 2500 error model, and uniform amplicon coverage produced by the ARTIC v5.3 primers.
- **Sample design.** One hundred independent pooled samples were simulated, each containing $2\times 10^{6}$ reads. Lineage proportions were specified a priori to cover the full spectrum from samples with a unique lineage to multi-lineage mixtures. Scenarios include balanced mixtures, as well as one dominant lineage accompanied by minor component, thus testing robustness in challenging abundance ranges.

This in silico benchmark is deliberately distinct from the spike-in dataset: it avoids wet-lab variability, supplies absolute truth for every mutation site, and permits systematic evaluation across (i) extreme abundance skews, (ii) closely related lineages, and (iii) fixed total read counts. Subsequent sections use these data to quantify both accuracy and resource usage of VaRaPS and competing tools.

### Protocol of the experiment

The experimental protocol consisted of two main phases: (1) constructing the lineage/mutation profile matrix *M* and (2) running the original implementations and VaRaPS on the simulated and spike-in mixtures validation datasets.

The first step involved building the lineage/mutation profile matrix *M* using data from GISAID [14]. The focus was on complete, high-coverage SARS-CoV-2 sequences from Europe collected between January 28, 2020 and January 7, 2024. A total of 118,228 sequences met the stringent selection criteria, which included completeness (>29,000 nucleotides), low ambiguity (<1% undefined bases), and high quality (<0.05% unique amino acid mutations).

The Nextclade tool was employed for clade assignment and mutation calling against the official hCoV-19 Reference Sequence from GISAID (hCoV-19/Wuhan/WIV04/2019 (WIV04)). A custom Python script then integrated the data into the matrix *M*, retaining a selection of VOC (variant of concern), VOI (variant of interest), and VUM (variant under monitoring) and mutations that pass a filter where their frequency is more than 0.1 in at least one lineage. The final matrix consisted of 31 lineages and 438 mutations. To ensure a fair comparison between the original implementations and VaRaPS, *M* was reformatted as needed for compatibility with each deconvolution method while preserving the underlying lineage and mutation frequency information.

The second phase involved applying the original codebases of Freyja [15], LCS [16], and VirPool [17], as well as their re-implementations in VaRaPS, to the 383 BAM files in the validation dataset from Karthikeyan et al. [15] and on the simulated dataset described in Sect. “Validation data”.

For the VaRaPS analysis, variant calling was performed on each BAM file separately, extracting mutation information at the read level. Subsequently, the re-implemented versions of Freyja, LCS, and VirPool within VaRaPS were applied to estimate the lineage proportions $\varvec{\pi }$ in each sample, incorporating an error rate parameter $\alpha$, fixed to 0.01, which is the default value considered in VaRaPS. Note that the choice of $\alpha$ has a surprisingly negligible impact on the estimated lineage proportions.

To manage the computational cost and time of the original VirPool implementation, downsampling was employed on spike-in mixture data. For five spike-in mixtures data files, the original VirPool method was applied without downsampling to establish a baseline for computation time. For the remaining spike-in mixtures data files, a downsampling factor of 20 was applied, or a minimum read count of 50,000 was set, whichever was higher, to accommodate smaller files. This approach ensured that the computational demands of the original VirPool method were manageable while preserving sufficient data for meaningful analysis.

Comparison of the estimated lineage proportions $\hat{\varvec{\pi }}$ with the known proportions in simulated and synthetic mixtures allows the method to be evaluated for accuracy and precision.

### Results of the experiments

#### Precision of the deconvolution results

***Precision results on the spike-in mixtures dataset***

**Fig. 1:**
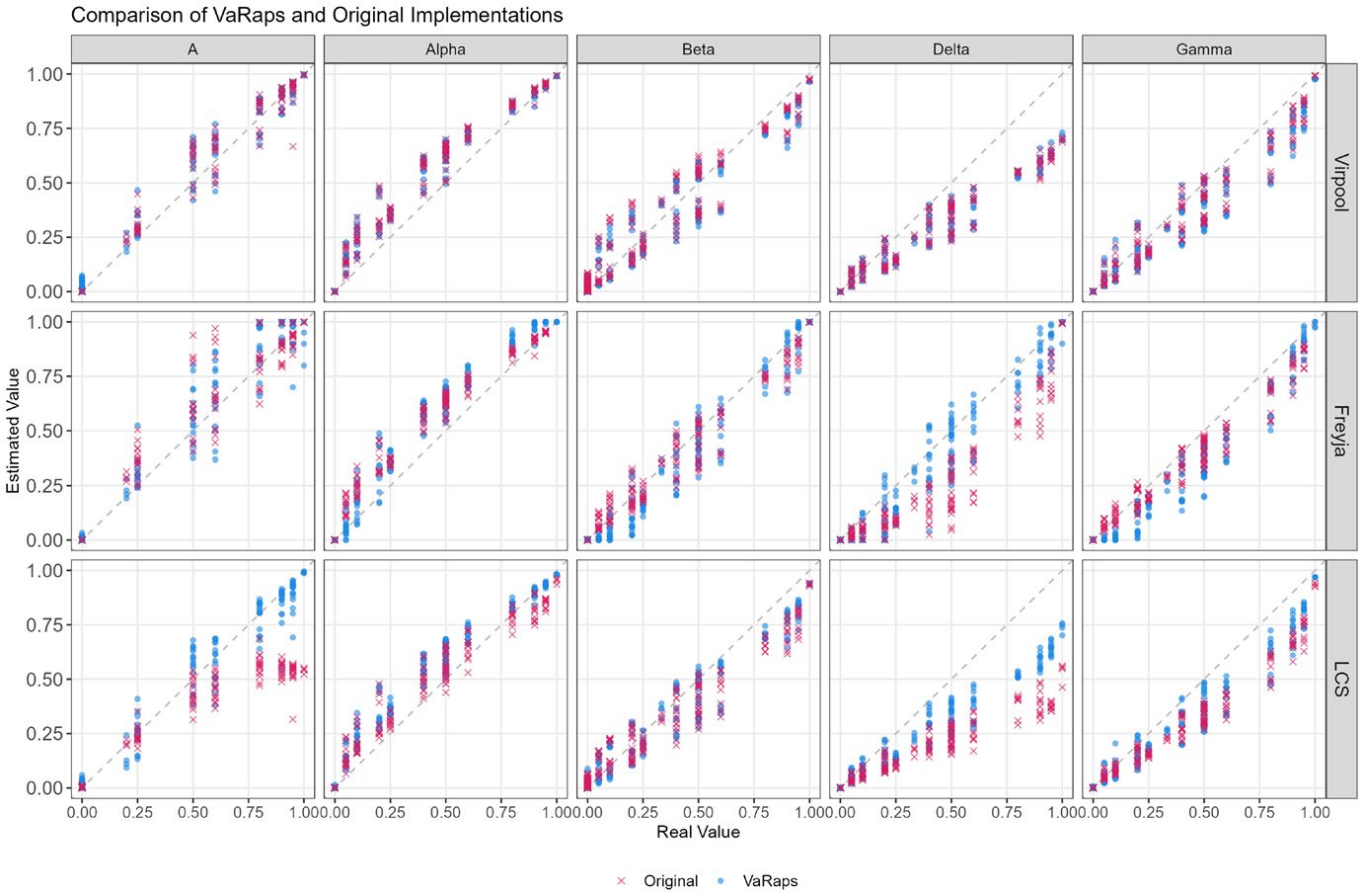
Precision comparison in lineage estimation between original implementation codebases (red) and VaRaPS re-implementations (blue) of Deconvolution Methods (Freyja, LCS, Virpool), against actual lineage proportion values, based on sequencing data from spike-in mixtures of five key SARS-CoV-2 lineages from Karthikeyan et al. [15]

Figure 1 provides a comparative assessment of the precision of lineages estimation between the original implementations and VaRaPS re-implementations of Freyja, LCS, and Virpool deconvolution methods on the real spike-in mixtures dataset. In a precise method, the data points would cluster tightly along the diagonal line representing perfect agreement between the estimated and actual values.

The original implementations of Freyja and Virpool exhibit high precision, as indicated by the concentration of points along the diagonal line. However, the LCS method shows a slight deviation from the diagonal, suggesting some bias in estimating lineage proportions.

In contrast, the VaRaPS re-implementation demonstrates a marked improvement in LCS precision, with data points more closely aligning with the diagonal line compared to its original implementation. This is mainly due to the sum-to-one constraint added to the estimation of proportions. Freyja and Virpool maintain their high precision in the VaRaPS re-implementation.

**Fig. 2:**
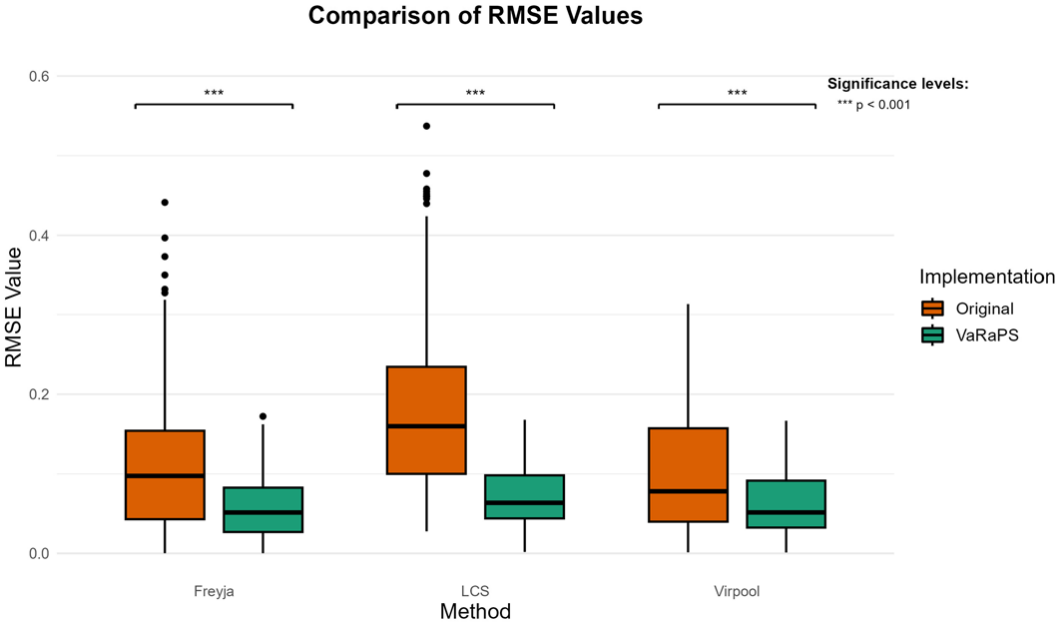
Boxplot comparison of Root Mean Square Error (RMSE) values for lineage proportion estimation using Virpool, Freyja, and LCS methods. The comparison is made between the original implementations of the methods (orange) and our re-implemented versions within the VaRaPS package (green). Lower RMSE values indicate higher accuracy. Outliers are represented by black dots. Statistical significance levels (*** $p < 0.001$) are shown above each method comparison, indicating highly significant differences between the original and our implementations

The comparison highlights the overall precision of the VaRaPS re-implementation. This is consistent with results obtained computing Root Mean Square Errors (RMSE) of lineage proportion estimations versus ground truth values for both the original implementations and the VaRaPS re-implementations of the Freyja, LCS, and Virpool methods, illustrated in Fig. 2.

The boxplots in Fig. 2 illustrate the RMSE values for each method, comparing the original implementations to those re-implemented within VaRaPS. Lower RMSE values indicate more accurate estimations. Our re-implemented versions consistently show lower RMSE values across Virpool, Freyja, and LCS. This improvement is statistically significant ($p < 0.001$) for all three methods:

- For Virpool, the Wilcoxon signed-rank test revealed a significant difference ($p < 2.81 \times 10^{-54}$) between the original and our implementation, with a median RMSE reduction of 0.0275.
- Similarly, Freyja showed a significant enhancement ($p < 6.68 \times 10^{-30}$) with our implementation, demonstrating a median RMSE reduction of 0.0406.
- LCS exhibited the most dramatic improvement, with a significant difference ($p < 1.96 \times 10^{-59}$) and the largest median RMSE reduction of 0.0867.

While the LCS re-implementation showed the largest absolute decrease in median RMSE, the VaRaPS implementation of Freyja resulted in the lowest median RMSE overall. However, the re-implemented Virpool also achieved a similarly low median RMSE, suggesting both methods provide comparable high accuracy among those tested.

To further evaluate the relative performance among the VaRaPS-reimplemented methods, pairwise statistical comparisons were conducted using the Wilcoxon signed-rank test on the RMSE values across the 383 independent samples.

This analysis revealed statistically significant differences in accuracy between LCS and the other two methods. Specifically, both VaRaPS Virpool (Wilcoxon signed-rank test, $p < 3.6 \times 10^{-31}$) and VaRaPS Freyja (Wilcoxon signed-rank test, $p < 3.5 \times 10^{-8}$) demonstrated significantly lower RMSE values, indicating higher accuracy, compared to the VaRaPS LCS implementation.

However, no statistically significant difference in RMSE was detected between the VaRaPS Virpool and VaRaPS Freyja implementations (Wilcoxon signed-rank test, $p = 0.31$). This suggests that within the VaRaPS framework and on this (relatively short-read) sequencing data, Virpool and Freyja achieve a comparable level of high accuracy, both significantly surpassing that of LCS based on the RMSE metric in this dataset.

Furthermore, the re-implemented versions display a notably reduced variance (indicated by the smaller interquartile ranges in the boxplots) compared to their original counterparts, implying more consistent and reliable performance.

### Computational performence

All the results of this section are obtained with AMD Ryzen 7 5800 H processor (3.2 GHz). They are all produced from the simulated dataset.

***Computational time performance of deconvolution algorithms***

**Fig. 3:**
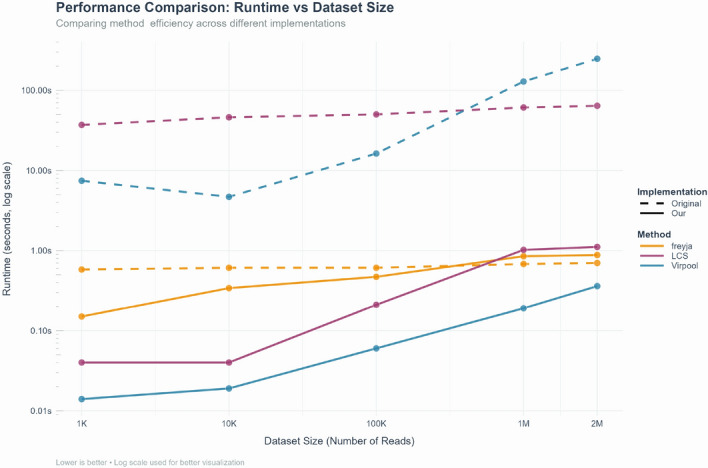
Optimization time comparison of lineage proportion estimation methods Virpool, LCS, and Freyja. The computational efficiency of the original implementations (dotted lines) are compared with our re-implemented versions within the VaRaPS package, with optimization time measured in seconds on a logarithmic scale

Figure 3 quantitatively compares the runtimes between the original implementations and our implementations of Virpool, LCS, and Freyja. It shows that our VaRaPS reimplementations markedly improve computational efficiency while preserving, or even enhancing, scalability with data file size.

For Virpool, the runtime drops from 247.15 to 0.36 s at 2 M reads (about $687\times$ faster) and exceeds $500\times$ acceleration at 1k reads as the large constant overhead of the original code is removed. LCS benefits even more from this reimplementation: 64s runtime is reduced to 1.11s at 2 M reads ($\sim 58\times$ faster) and accelerations are above $900\times$ for data files of 1k reads or fewer. Freyja, already highly optimized, remains sub-second for all inputs; our version is faster for small files (up to $4\times$ at 1k reads) but marginally slower (by $\le$ 0.2s) for the largest inputs.

An important observation is that the VaRaPS implementation of Virpool is even faster than the more direct approaches of Freyja and LCS. This is due to two key factors: first, at the initial optimization step, we group all identical reads (identified solely by mutation positions) and use weights based on their repetition counts–for instance, a file of 2 M reads reduces to only about 3000 unique "reads" with high repetition. Second, the VaRaPS Virpool optimization uses an explicit, analytically derived formula (see Appendix), whereas the VaRaPS implementations of Freyja and LCS rely on numerical optimization via scipy.optimize.

Across five orders of magnitude in input size, the VaRaPS curves follow an almost straight line on the log–log plot, confirming near-linear time complexity. Consequently, multi-million-read wastewater samples can now be deconvolved in seconds on a standard workstation, turning what was once a computational bottleneck into a routine step of the analysis pipeline.

**Fig. 4:**
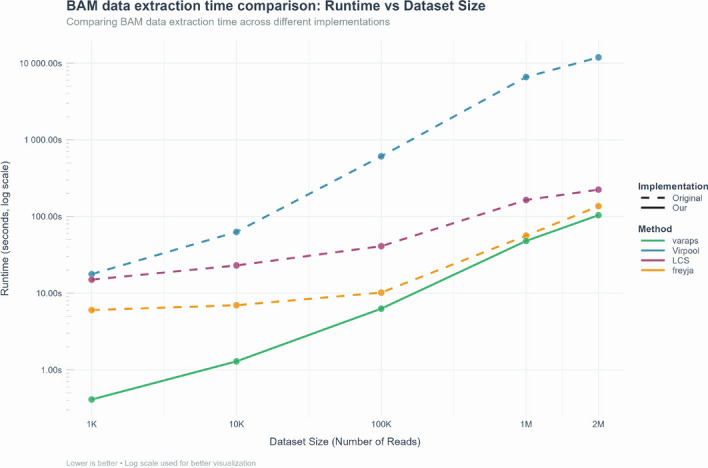
BAM data extraction time comparison across Virpool, LCS, and Freyja methods. The graph demonstrates the time efficiency of data extraction in the original implementations (dotted lines) against our implementations (solid green line), with extraction time presented in seconds on a logarithmic scale versus dataset size (number of reads)

When comparing the BAM data extraction times (Fig. 4), the original implementation of Virpool required more than 11, 894 seconds to treat a sequencing file with two million reads, while our implementation reduced this time to 104 seconds (thus a 114 speed factor). Similarly, the extraction time for LCS decreased from 224 s in the original to 104 s in our version (thus a 2.2 speed factor). For Freyja, the extraction time is reduced from 136.46 s in the original to 104 s in our implementation (thus a 1.3 speed factor). In the cases of Freyja and LCS, the extraction times were not expected to be significantly reduced since such methods necessitate summarized data (mutation counts for LCS or mutation frequencies for Freyja) and thus basic extraction methods are sufficient. However, VaRaPS extraction times were similar to theirs for big sequencing files (e.g., 2 M reads) and significantly reduced compared to theirs for small sequencing files.

These reductions in extraction times are very useful for handling large-scale sequencing data.

#### Peak RAM memory usage comparison

**Fig. 5:**
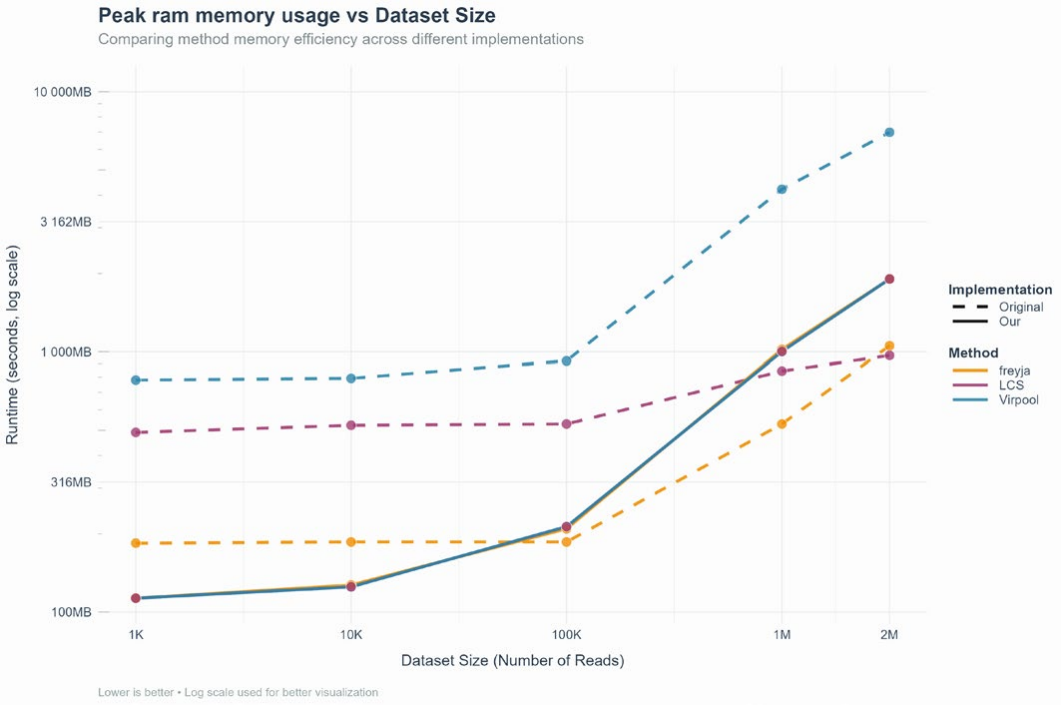
Peak RAM usage across data files sizes for Freyja (orange), LCS (magenta) and VirPool (blue). Dashed lines show the original codebases, solid lines the VaRaPS versions (log–log scales; lower is better)

Figure 5 shows maximum resident RAM required by the three lineage-deconvolution workflows as a function of input depth (1 $\times$ $10^{3}$–2 $\times$ $10^{6}$ reads; logarithmic axes).

The VaRaPS version consistently lowers the footprint of VirPool method, from 780 MB$\rightarrow$113 MB at 1 k reads ($-86\%$) to 6.98 GB$\rightarrow$1.90 GB at 2 M reads ($-73\%$).

In the case of LCS method, for data sets $$\le $$$$10^{5}$$ reads the VaRaPS implementation is more economical (529 MB$$\rightarrow $$213 MB at $$10^{5}$$ reads, $$-60\%$$), but at higher sizes its use of explicit sparse matrices raises peak RAM to $$\approx $$1.90 GB (original: 0.97 GB). However, the increase is modest relative to the $$\ge $$50-fold acceleration reported in the previous subsection.

In the case of the Freyja method, a similar crossover is observed: VaRaPS reduces RAM at 1 k reads (184 MB$\rightarrow$113 MB, $-39\%$) yet rises to 1.90 GB at 2 M reads (original: 1.05 GB).

Notably, the peak RAM usage under VaRaPS is identical across all three methods for each data file size. This occurs because the measurements include both the BAM data extraction and optimization steps; the BAM extraction step is shared among the methods and dominates RAM usage (e.g., peaking at 1903 MB for 2 M reads, compared to 442 MB for the optimization step alone).

Taken together, the measurements show that (i) the VirPool re-implementation substantially improves memory scalability across the entire range examined, and (ii) the LCS and Freyja re-implementations exchange a limited increase in RAM at very large data volumes ($<2$ GB on a standard workstation) for the runtime advantages required in high-throughput surveillance.

***Downsampling and computational efficiency***

**Fig. 6:**
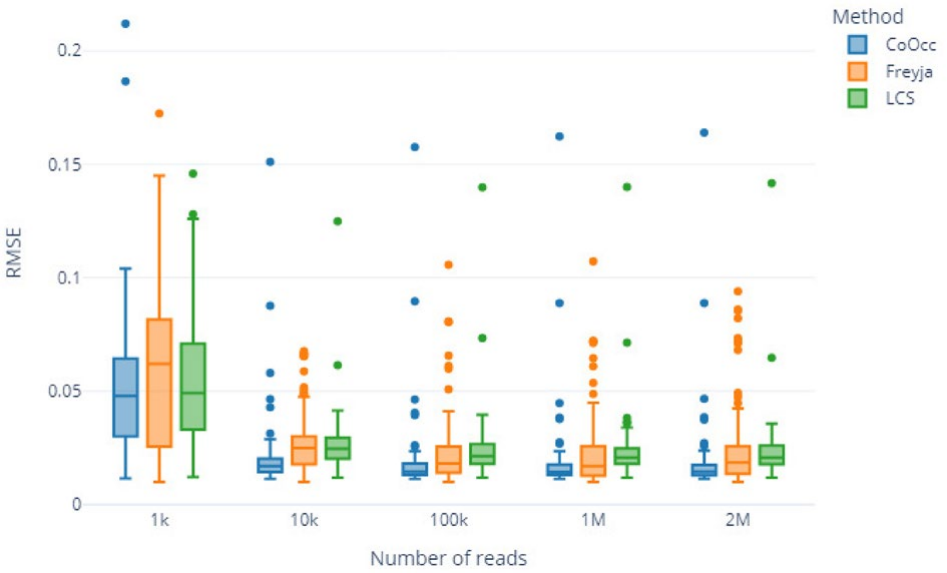
Comparison of Root Mean Square Error (RMSE) values for lineage proportion estimation using original sequencing files with two million reads and downsampled (down to 1, 000 reads) sequencing files across Virpool, Freyja, and LCS methods (VaRaPS)

To assess the impact of downsampling on estimation accuracy and computational efficiency, we compared the performance of all methods using both the original simulated sequencing data (read count of $2\times 10^6$) and downsampled data (down to 1, 000 reads per sample). Figure 6 illustrates the RMSE distributions for both scenarios across Virpool, Freyja, and LCS methods in VaRaPS.

While the downsampled versions show notably increased RMSE values from 10, 000 to 1, 000 reads, the overall accuracy remains robust from $2\times 10^6$ to about 10, 000 reads. This suggests that the downsampling approach effectively preserves the essential information required for accurate lineage proportion estimation including at the rate applied to the data for running the original VirPool method, which never retains less than 50, 000 reads.

This robustness extends to challenging cases with low-abundance lineages.

**Fig. 7:**
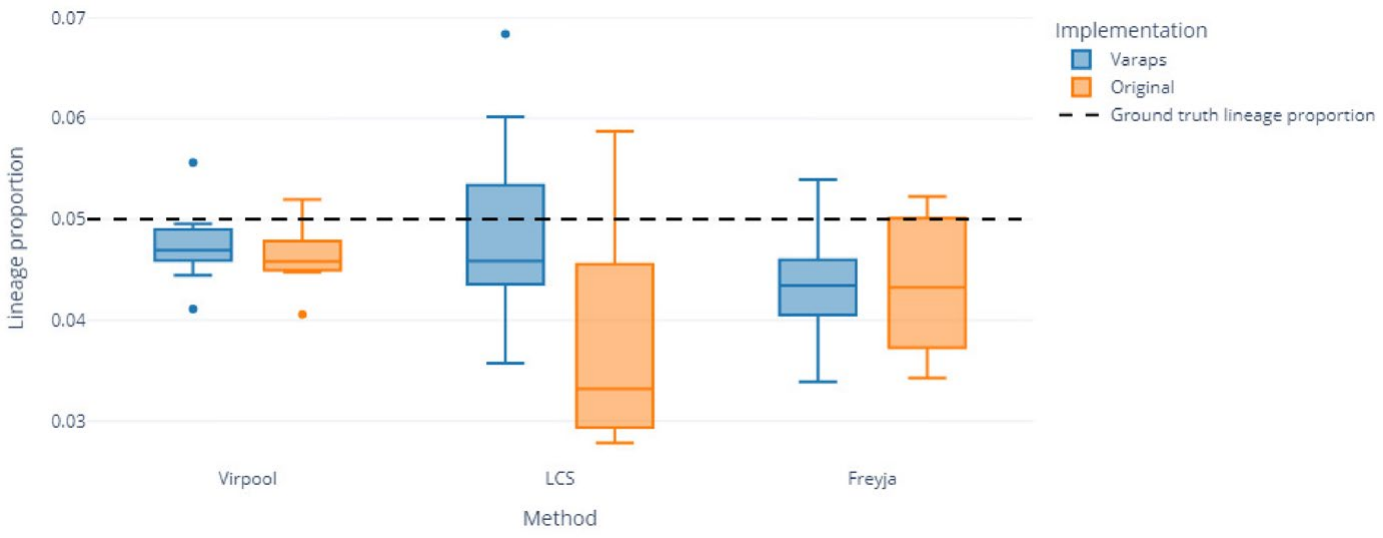
Estimation of the minor lineage proportion (0.05) from data simulated with a minor lineage and one or several other lineages, for original implementation codebases (orange) and VaRaPS re-implementations (blue) of deconvolution methods (Freyja, LCS, Virpool). Results are shown for downsampled files (100k reads from original 2 M reads)

Figure 7 highlights the precision of lineage estimation at low abundance (0.05) for one of the simulated lineages, comparing VaRaPS and original implementations across methods. VaRaPS consistently slightly outperforms original methods at this prevalence level, with tighter estimated values around the true proportion, reduced estimation variance, and bias correction, notably in LCS, where VaRaPS eliminates the systematic underestimation seen in the original implementation. In this simulation setting, also characterized by slightly longer reads (read length of 250bp versus 151bp in Spike-in mixtures datasets), VirPool achieves the highest precision on low-abundance lineages by taking advantage on read-level mutation co-occurrence, which provides additional information compared to Freyja’s frequency-based approach and LCS’s mutation count-based model. In particular, results on the full dataset before subsampling (2 M reads) were very similar, indicating that downsampling to 100k reads has minimal impact on these performances in the low-abundance case.

### Results discussion

The comprehensive evaluation of the VaRaPS package through a series of methodical comparisons has demonstrated its substantial contribution to the field of viral lineage analysis. The precision in lineage estimation has been validated on both realistically simulated and real spike-in data and significantly refined, as evidenced by the close alignment of the estimated values to the actual proportions in our implementation and reduced RMSE in lineage proportion estimation. This precision is fundamental for accurate surveillance and management of viral outbreaks [6, 26].

The quantitative analysis of optimization and data extraction times further reveals that the VaRaPS package provides a significant gain in computational speed. The optimization process for lineage proportion estimation methods has been expedited dramatically, notably with the original Virpool method’s optimization time of over three minutes (on sequencing data with two million reads) reduced to mere milliseconds and similar enhancements observed for LCS method. Data extraction from BAM files, a typically time-intensive process, has also seen remarkable improvements, with reductions from hours to minutes.

The possibility of downsampling offered by VaRaPS, with no significant impact on deconvolution results even when there is a low abundance lineage (at a frequency around $5\%$), provides additional savings in terms of resources and computing time, in addition to those achieved by the optimized implementation within VaRaPS.

Those optimizations are particularly valuable for large-scale surveillance efforts, where rapid analysis of numerous samples is necessary for timely public health responses.

Interestingly, while both VaRaPS Virpool and VaRaPS Freyja significantly outperformed VaRaPS LCS in terms of accuracy, our statistical analysis indicated no significant difference in performance between VaRaPS Virpool and VaRaPS Freyja on the real (short-read) spike-in mixtures dataset used in this study (Bonferroni-corrected $p = 0.94$). This finding warrants consideration in light of Virpool’s design, which leverages information about the co-occurrence of mutations within single sequencing reads-a feature not utilized by Freyja or LCS. Analysis of the sequencing data revealed that the 151 bp read length predominantly resulted in reads covering either zero or only one informative mutation site. This inherent limitation directly hindered Virpool’s capacity to leverage mutation co-occurrence information within individual reads in this real data experimental setup. The complementary study on simulated data confirms the potential of co-occurence-based deconvolution methods can be revealed even with sequencing data produced with the Illumina technologie (thus with rather short reads compared to sequencing data that would be obtained from Oxford Nanopore-like technologies), since VirPool-VaRaPS outperformed Freyja’s (and LCS’s) on those simulated data (with a read length of 250bp).

Future applications using longer-read sequencing technologies could potentially allow Virpool to better exploit its co-occurrence detection capabilities and might reveal a more distinct performance advantage.

## Conclusion and perspectives

The development and comprehensive evaluation of VaRaPS (Variants Ratios from Pooled Sequencing) represent a significant advancement in the field of bioinformatics, specifically in the context of SARS-CoV-2 lineage analysis from wastewater sequencing data. This Python package successfully addresses the limitations of existing tools, such as Freyja [15], LCS [16], and VirPool [17], by offering improved accuracy, computational efficiency, and a unified framework for data processing and variant calling.

The comparative analysis of VaRaPS against the original implementations of Freyja, LCS, and VirPool, using simulated and synthetic datasets [15], demonstrates its superior performance in estimating lineage proportions. The precision of VaRaPS’s re-implemented methods, particularly the enhanced accuracy of the LCS method, underscores its reliability in providing actionable epidemiological insights.

Moreover, the substantial gains in computational speed, as evidenced by the dramatic reductions in optimization and data extraction times, position VaRaPS as a scalable solution for large-scale genomic surveillance efforts [6, 27] even more so since it significantly reduces the peak RAM memory required when using the most advanced method (VirPool).

VaRaPS was furthermore applied to real-world settings, such as SARS-CoV-2 sequencing in wastewater from the Obépine Network, for both variant calling uses [28] and variants proportions estimation, and gave outputs corresponding to the variants proportions that could be computed from clinical samples gathered in GISAID at the same period.

As the global community continues to navigate the challenges posed by the ongoing COVID-19 pandemic and the emergence of novel SARS-CoV-2 lineages [28], tools such as VaRaPS will play an increasing role in guiding evidence-based interventions and policies.

The increasing adoption of long-read sequencing technologies, such as those offered by Oxford Nanopore, presents a promising future direction. Longer reads inherently contain more information about the co-occurrence of mutations on the same viral template which could significantly enhance the resolution and accuracy of methods like Virpool within the VaRaPS framework, potentially allowing for finer differentiation between closely related lineages [29]. In the wastewater matrix, improvements from longer reads may nevertheless be limited. Indeed recent publications showed that SARS-CoV-2 genomes extracted from wastewater are largely fragmented, a process that depends on the nature of the matrices, the extraction steps, and the conservation of the samples [30, 31].

When sequencing technology involves the production of paired-end reads, another approach in this direction would be to use this pairing information. The next version of VaRaPS, currently under evaluation, will likely include the option to do so by merging the information of all reads that carry the same identification number and then treating them as if they were a single read, thus doubling their length and opening up a new avenue for improving the performance of our deconvolution tool.

Although our evaluation focused on SARS-CoV-2, VaRaPS is applicable to other pooled sequencing data of viruses, provided that lineage definitions are available in the form of a mutations frequencies matrix. This flexibility is enabled by the option to load custom lineage matrices, allowing users to adapt the framework to other viral pathogens and lineage nomenclatures, including those developed for future epidemic surveillance (such as the framework proposed by Mc Broome and co-autors [32]). This approach could for instance be used for other viruses frequently detected in wastewater in the form of mixtures. This is particularly true for variable RNA viruses, such as noroviruses, enteroviruses, hepatitis E virus, arboviruses, human and avian influenza viruses for example [33] as well as for DNA viruses that are genetically stable, but whose several variants circulate concomitantly, such as human papillomaviruses [34].

## Availability and requirements

- **Project name:** VaRaPS: Variants Ratios from Pooled Sequencing.
- **Project home page:** https://pypi.org/project/VaRaPS/.
- **Operating systems:** Platform independent.
- **Programming language:** Python.
- **Other requirements:** Python 3.8 or higher and numpy, scipy, pandas, pysam, cigar, tqdm.
- **License:** GPL-3.0 license.
- **Any restrictions to use by non-academics:** GPL-3.0 license.

## Figures and Tables

**Algorithm 1:**
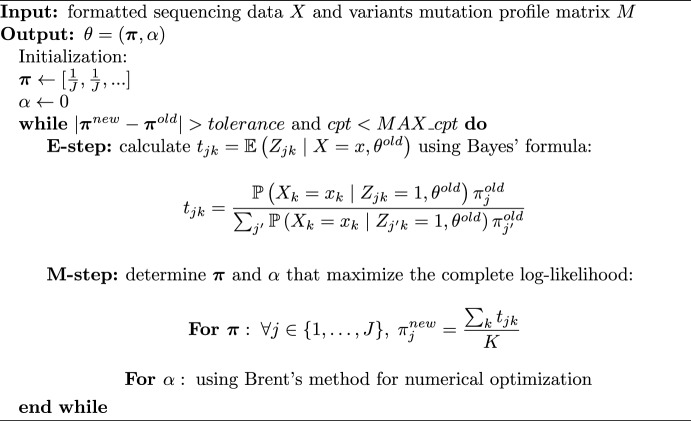
Expectation-Maximization (EM) Algorithm

## Data Availability

Spike-in sequencing data are provided by and available via Google cloud https://console.cloud.google.com/storage/browser/search-reference_data . Consensus sequences from clinical and wastewater surveillance are all available on GISAID. The simulation files generated for this experiment are available at https://app.filen.io/#/f/6b6fcec3-8bb7-42d2-b2ba-1c39251b50d0%23IPeg7H6q748wewhEa55Wdjb1Lj4s8u1w The SARS-CoV-2 variant mutations profile used for this work is available at https://github.com/hacen-ai/Varaps-data/blob/main/Variant-mutation-profile-matrix.csv

## Abbreviations

SARS-CoV-2: Severe acute respiratory syndrome coronavirus 2
WGS: Whole genome sequencing
RT-PCR: Reverse-transcriptase polymerase chain reaction
RNA-seq: RNA sequencing
EM: Expectation–Maximisation
WWTP: Wastewater treatment plant
GISAID: Global Initiative on Sharing Avian Influenza Data
BAM: Binary alignment map
CRAM: Compressed reference-oriented alignment map
SAM: Sequence alignment map
CIGAR: Compact idiosyncratic gapped alignment report
VOC: Variant of concern
VOI: Variant of interest
VUM: Variant under monitoring
RMSE: Root mean square error
GPL: GNU general public license
ISCD: Institut des Sciences du Calcul et des Données
ANRS-MIE: Agence Nationale de Recherche sur le Sida et les hépatites virales - Maladies Infectieuses Émergentes
OBEPINE: Observatoire Épidémiologique dans les Eaux Usées

## Code availability

Varaps is open sourced and hosted publicly on GitHub (https://github.com/hacen-ai/varaps) and is also accessible as a package via pypi (https://pypi.org/project/VaRaPS/).
